# Filling Teeth

**Published:** 1868-11

**Authors:** Gam’L Jackson


### FILLING TEETH.

BY GAM'L JACKSON.

THE inestimable superiority of the natural teeth compared with the best artificial ones being conceded by all intelligent persons, and the operation of plugging decayed teeth being the commonest means employed by the Dentist for their preservation, it is a matter of first importance that he should be thoroughly acquainted with the materials used for this purpose, and the best methods of applying them. He is bound to be thus qualified on account of the responsibility he assumes in accepting the care of his patients' teeth, and for his own current satisfaction and future peace of mind. When a Dentist makes a plug that he knows is up to the present standard, he feels that he has made a contribution to science, and thus secured the grateful remembrance of a fellow being, and he also feels again that peculiar satisfaction associated with success in whatever we undertake.

The subject of filling teeth, important as it is, might appear to be already exhausted; but so long as Dentists, who evidently read the journals, ask "how to fill a tooth," or recommend "the fractured end of a broken bur or excavator, as an excellent drill," or persist in recognizing amalgam as efficacious, no apology will be necessary for writing on this subject, even though what may be written may not be characterised by entire originality.

Leaving pathalogical conditions to other writers I shall discuss only the mechanical principles upon which teeth are filled, confining myself in this article to cavities of the representative class, in which all of the walls are still remaining, and strong enough to resist moderate outward pressure.

All cavities opening on the coronal surface, and others which are very small, should be filled with adhesive gold foil and sponge gold, for first class work, while those opening on the other surfaces of the teeth are not exposed to direct wear, are best filled, as a rule, with soft foil. This choice of materials is made because a plain cavity can be filled in less time and with less labor with soft foil; it is sufficiently hard to last a life time when not exposed to direct wear; a more perfect adaptation can be made of it to the walls of the cavity, particularly at the margin, where impermeability is indispensable; and the integrity of the plug does not depend upon its being kept absolutely dry during its introduction. Adhesive foil and sponge gold are chosen for the coronal surface because hardness is here an essential quality, and the conditions are more favorable for introducing them efficiently.

A model cavity is one with a level floor and walls parallel to each other or slightly convergent. As such cavities are hardly ever naturally produced, it is necessary that the sharp edges of those having too convergent walls, and the surface of those of opposite character should be cut away so as to at least approximate the model shape. In preparing those cavities having quite thin walls the edges should only be cut away sufficiently to leave them smooth and firm, and the concave walls should be made perpendicular by the application of blocks or pellets of foil. In cutting these edges I use instrument shaped like hoe and hatchet excavators, but with all three of the edges of the blades sharpened for cutting.

In preparing gold or tin foil, I use Dr. McClellan's foil crimper, but made of wood instead of tin, with square brass rods rivited on to two opposite borders of each side of both boards. By this arrangement, and for large blocks crimping two leaves together, four sizes may be made. After the foil is crimped into compact bars they are cut into blocks of four or five different lengths. The best method is to hold the bar on the smooth surface of lead or box-wood, using a razor blade in a permanent handle. The block should then be compressed again in the direction that they were crimped. This is readily done between a pair of plugging tweezers, or a number may be finished at once between the block on which they are cut and one of the boards of the crimper. The blocks thus prepared are kept most conveniently for use in a shallow compact box, each size being kept by itself. One apartment of the box should be kept for finishing-cylinders, made by first annealing blocks to make them slightly adhesive, and then compressing them to a cylindrical shape between the tweezers, the blades of which, for this purpose, should be properly grooved back some distance from the points.

To illustrate the use of these blocks, I will suppose that an apple barrel is to be filled so as to secure the results required in filling a tooth, and that the material is thin oil cloth twenty feet square. One method would be to twist or fold the cloth into ropes or strips, and fold them longitudinally into the barrel, making each fold to reach the bottom and project slightly from the top. When full, a tapered instrument would be forced between the folds, and the space filled with folds of cloth. Another method would be to cut the cloth into strips slightly wider than the depth of the barrel, and having rolled them into cylinders, place them, and finish as in the first method. Still another method would be to prepare the cloth according to the above described method for foil, and finish as in the first method, using finishing-cylinders more tightly rolled than the others. An important objection to the second method is the difficulty and uncertainty of obliterating the triangular spaces between the cylinders and the walls of the barrel. This difficulty exists, but in a less degree, with the folded strips in the first method, and in a much less degree with the twisted ropes. But the extreme difficulty of folding ropes of foil into cavities in the teeth is a serious objection to this method. Blocks are much more easily managed, and equally good results uniformly attend their use. Their structure is more favorable to the production of perfect plugs on account of the greater lateral mobility of the folds composing them, and the ease with which a pointed instrument may be made to penetrate them at any part. It is important, however, that in pressing them to the walls, as they are severally placed in the cavity, their peculiar structure should be kept in view.

In filling the barrel with blocks of cloth, one as long as the barrel and thick enough to nearly fill it, should be first placed in. An instrument with a broad flat blade turned at right angles with the shaft should then be placed against one of the corners of the block, and the pressure applied diagonally. The folds would thus be made to slide on each other, and imbricate along the walls. Other smaller blocks should then be introduced and compressed in the same way to fill all the space. An instrument tapered to a sharp point should then be forced to the bottom, near the last block, and the hole enlarged with a similar instrument, but with less taper and a blunt point. Into this hole a finishing-cylinder should be forced, and the process continued until the plug is well wedged in. The same principles should be observed in filling teeth. If the structure of the block, and the effects of pressure applied to them at different angles are studied, the results will be satisfactory. Care must be taken to keep all of the blocks to the bottom of the cavity, consolidating them as they are introduced, rather than in finishing; not neglecting, however, to go over the surface of the plug with a small plugger, and with the plugging pliers wherever safe.

